# Krüppel Like Factor 4 Promoter Undergoes Active Demethylation during Monocyte/Macrophage Differentiation

**DOI:** 10.1371/journal.pone.0093362

**Published:** 2014-04-02

**Authors:** Manjula Karpurapu, Ravi Ranjan, Jing Deng, Sangwoon Chung, Yong Gyu Lee, Lei Xiao, Teja Srinivas Nirujogi, Jeffrey R. Jacobson, Gye Young Park, John W Christman

**Affiliations:** 1 Section of Pulmonary, Allergy, Critical Care and Sleep Medicine, The Ohio State University Wexner Medical Center, Davis Heart and Lung Research Institute, Columbus, Ohio, United States of America; 2 Section of Pulmonary, Critical Care, Sleep and Allergy, College of Medicine, University of Illinois at Chicago, Chicago, Illinois, United States of America; 3 Institute for Personalized Respiratory Medicine, Department of Medicine, University of Illinois at Chicago, Chicago, Illinois, United States of America; University of Texas Southwestern Medical Center, United States of America

## Abstract

The role of different lineage specific transcription factors in directing hematopoietic cell fate towards myeloid lineage is well established but the status of epigenetic modifications has not been defined during this important developmental process. We used non proliferating, PU.1 inducible myeloid progenitor cells and differentiating bone marrow derived macrophages to study the PU.1 dependent KLF4 transcriptional regulation and its promoter demethylation during monocyte/macrophage differentiation. Expression of KLF4 was regulated by active demethylation of its promoter and PU.1 specifically bound to KLF4 promoter oligo harboring the PU.1 consensus sequence. Methylation specific quantitative PCR and Bisulfite sequencing indicated demethylation of CpG residues most proximal to the transcription start site of KLF4 promoter. Cloned KLF4 promoter in pGL3 Luciferase and CpG free pcpgf-bas vectors showed accentuated reporter activity when co-transfected with the PU.1 expression vector. *In vitro* methylation of both KLF4 promoter oligo and cloned KLF4 promoter vectors showed attenuated *in vitro* DNA binding activity and Luciferase/mouse Alkaline phosphotase reporter activity indicating the negative influence of KLF4 promoter methylation on PU.1 binding. The Cytosine deaminase, Activation Induced Cytidine Deaminase (AICDA) was found to be critical for KLF4 promoter demethylation. More importantly, knock down of AICDA resulted in blockade of KLF4 promoter demethylation, decreased F4/80 expression and other phenotypic characters of macrophage differentiation. Our data proves that AICDA mediated active demethylation of the KLF4 promoter is necessary for transcriptional regulation of KLF4 by PU.1 during monocyte/macrophage differentiation.

## Introduction

Myeloid cell differentiation is controlled by a complex circuitry of lineage specific transcription factors and the role of the Ets family transcription factor PU.1 in myeloid lineage specification is well documented [1], [2]. PU.1 expression levels critically determine the specification of myeloid and common lymphoid progenitors [3] and knock down of PU.1 in mice results in defective development of macrophages and granulocytes [4], [5]. Interestingly, high levels of PU.1 support macrophage development whereas low levels support the production of granulocytes [6]. Also, co-operative or antagonistic interactions of PU.1 with other transcriptional factors decides the cell fate towards erythrocytic, B-cell, mast or dendritic cell lineages [7], [8]. The importance of Krüppel like factor 4 (KLF4) in inflammatory monocyte differentiation *in vivo* and also in early monocyte development was identified by Alder et al [9] and Feinberg et al [10]–[11]. X-ray crystal studies of KLF4 protein revealed that the deletion of the two C-terminal zinc fingers lead to deficiency of KLF4 expression resulting in macrophage self renewal and defective differentiation [12]. Recent genome wide studies on epigenetic and transcription factor profiling in different cell types correlated the lineage specific transcription factor binding with changes in epigenetic marks like histone acetylation/methylation and DNA methylation at the target gene promoters that potentially affect the cell fate.

DNA methylation is a dynamic epigenetic change that regulates gene expression by controlling accessibility of DNA to transcription factor binding. DNA methylation marks are lost either by passive or active mechanisms where passive demethylation occurs in actively replicating cells and active demethylation is observed in non replicating cells. The role of mammalian DNA methylating enzymes DNA methyl transferase 3A/3B and DNMT1 in maintaining DNA metjhylation marks is well characterized whereas the mechanisms of demethylation have been elusive and it was believed that it occurs by a passive process during DNA replication. The process of active demethylation and exact mechanism is still under debate [13]–[16]. Initial studies reported that active DNA demethylation to be associated with DNA Base Excision Repair proteins like Thymine DNA Glycosylase (TDG), Methyl CpG binding Domain (MBD) and Growth Arrest and DNA Damage inducible (GADD45) proteins [17]–[20]. Activation Induced Cytidine Deaminase (AICDA) that deaminates methyl Cytosine was also reported to play a crucial role in active DNA demethylation [21]–[23]. Recent studies also advocated coupled action of GADD45α and TDG or Ten Eleven Translocation (TET) proteins followed by the deaminase AICDA for active demethylation [19], [20]. Data on generation of 5 methyl cytosine intermediates like 5 hydroxylmethyl cytosine, 5 formyl cytosine, 5 carboxyl cytosine that are further calalyzed by TDG via base excision repair or TET proteins indicate additional levels of regulation during the active demethylation [24]–[27]. Another interesting finding is that, the deaminase AICDA also alters subcellular localization of TET family proteins, which may possibly affect the TET mediated demethylation [28]. Despite the extensive efforts, direct evidence on the role of these protein factors in 5 mC base conversions is still incomplete and knock out mice studies are still inconclusive [21], [29]. In our studies to delineate the epigenetic mechanisms involved in PU.1/KLF4 dependent monocytic macrophage differentiation, we observed that expression of KLF4 is regulated by PU.1 at transcriptional level and this event is linked with active demethylation of KLF4 promoter. In the present study using non-proliferating PU.1 inducible myeloid progenitor cells and differentiating bone marrow derived macrophages (BMDM), we aimed to identify the regulation of KLF4 promoter demethylation during monocyte/macrophage differentiation and the mechanism/protein factor(s) involved in this process.

## Materials and Methods

### Cells

PU/ER(T) cell line was a gift from Dr. Harinder Singh, University of Chicago [30]. PU/ER(T) cells were grown in IMDM supplemented with 1% Glutamine, 1% β-mercapto ethanol, 10%FBS, 50 IU/mL Penicillin and 50 μg/mL Streptomycin and 5 ng/mL IL-3. Bone marrow cells were differentiated in to mature macrophages (BMDMs) from wild type and GADD45α mice in DMEM supplemented with 10% FBS, 50 IU/mL Penicillin and 50 μg/mL Streptomycin and 20 ng/mL recombinant mouse M-CSF added on Day 1, 3 and 5. Cells were allowed to differentiate up to 7 days in total. HEK 293 cells were cultured in DMEM F12 with 10% FBS and 50 IU/mL Penicillin and 50 μg/mL Streptomycin.

### Mice

Wild type C57 black6 mice were purchased from Jackson Laboratories. GADD45α knock out mice were a gift from Dr Jeffery Jacobson at the Institute of Personalized Respiratory Medicine, University of Illinois Chicago. All the animal experiments and procedures were conducted with protocols approved by the Institutional Animal Care and Use Committee (IACUC) of the University of Illinois at Chicago and The Ohio State University, Columbus.

### Antibodies and fine chemicals

Recombinant mouse M-CSF was purchased from R&D systems (416-ML/CF) and Recombinant mouse IL-3 was purchased from Invitrogen (#PMC0035). 4-Hydroxy tamoxifen (4-OHT) for cell culture was purchased from Sigma-Aldrich (H7904-5MG). Antibodies for flow cytometry analysis, PE rat anti–mouse CD 11b was purchased from BD Pharmingen (Cat# 557397) and PE anti–mouse F4/80 from eBioscience (Cat# 12-4801-82). Antibodies for immunoblotting of proteins were purchased from different companies as indicated: PU.1 (Cell Signaling Technology # 2266, Santacruz sc-352), KLF4 (AF3158 R&D systems; Santacruz sc-20691), AICDA (Abcam 59361; Santacruz sc-14680), GADD45α (sc-797), TDG (sc-22845), MBD4 (sc-365974), GATA-1 (sc-1234) and α-Tubulin (sc-8035). Genomic DNA was isolated using Promega genomic DNA isolation reagents (Nuclei lysis solution #A7943; Protein precipitation solution #A7953), Bisulfite conversion of methylated genomic DNA was carried out by Invitrogen MethylCode Bisulfite Conversion Kit (Cat# MECOV-50). CpG methyl transferase (*M. SssI*) was purchased from NEB (Cat# M0226S). Protein A-Sepharose, Protein A/G Sepharose (CL-4B) and Myc-tagged Immunoprecipitation kit was purchased from Pierce (#23625). Mouse monocyte enrichment kit was purchased from Stem Cell Technologies (Cat# 19761). All the primers and oligonucleotides were synthesized by IDT (Coralville, IA) and listed in Supplement S1. ChIP One Day Chromatin Immunoprecipitation kit was purchased from QIAGEN (Cat# 334471).

#### Vectors and Transfection Reagents

Myc tagged AICDA and FLAG tagged human KLF4 expression vectors were purchased from Origene (RC202949) and SABiosciences (DAM-603), respectively. Luciferase promoter probe vector pGL3 was purchased from Promega and pCpG free-basic vector containing mouse Alkaline phosphotase (mSEAP) reporter gene was purchased from Invivogen (#pcpgf-bas). AICDA and Control non specific shRNA vectors were purchased from Origene (TF515096). Transient transfection of PU/ERT cells with specified vectors was carried out using mouse macrophage Nucleofector kit (Lonza VPA-1009) and Amaxa Electroporation unit. cDNA vectors of AICDA, PU.1 and pGL3-KLF4 promoter or pcpgf-bas-KLF4 promoter were transfected in to HEK293 cells using Lipofectamine 2000 (Invitrogen #52887) or macrophage nucleofector kit.

### Western Blot Analysis

PU/ER(T) cells with and without Tamoxifen treatment or bone marrow derived macrophages from wild type or GADD45α knock out mice were harvested, and cell extracts were prepared in 1X RIPA buffer (Cell signaling Technologies #9806) supplemented with protease inhibitors. An equal amount of protein was analyzed by Western blotting for the protein of interest using its specific antibodies according to standard protocols.

### RT-PCR

Total cellular RNA was isolated from cells under different experimental conditions using QIAGEN RNeasy Plus Mini Kit (#74134) following the manufacturer's instructions. Reverse transcription was carried out with the Fermentas cDNA synthesis kit for RT-PCR using the supplier's protocol, and the cDNA was then used as template for PCR using primers (listed in Supplement S1, at least one of the primer was picked from exon/exon junction for mRNA expression assay) specific for mouse KLF4, GATA1, GATA2, AICDA and β-Actin. The PCR amplification was carried out using Applied Biosystems SYBR green reaction mix (#4309155) on Applied Biosystems Real Time PCR machine 7500 or Roche 480 PCR machine.

### Electrophoretic Mobility Shift Assay

Nuclear extracts of Tamoxifen treated PU/ER(T) or bone marrow derived macrophages during different differentiation stages were prepared and analyzed for DNA binding activity. 5′ biotin labeled KLF4 promoter oligo was incubated with 5 μg nuclear protein extract, resolved on 6% native Acrylamide gels using 0.5X TBE buffer. The protein DNA complexes from the gels were transferred on to Biodyne A Nylon membrane (#77015) and developed using North2South Chemiluminescent Developing reagent kit (#89880) from Pierce according to the supplier's instructions.

### Chromatin Immunoprecipitation (ChIP) Assay

PU/ER(T) or BMDM cells were subjected to appropriate treatments, cross linked using 1% formaldehyde for 10 min at 37°C and washed with PBS. Cross linking was stopped with 0.125 M glycine in PBS, cells were washed in PBS, centrifuged for 5 min at 1200 rpm, and pellets were processed using the buffers supplied in QIAGEN Epitect one day Chromatin Immunoprecipitation kit. Cell pellets were sonicated and pre-cleared using Protein A agarose supplied in the kit. Resulting Chromatin was Immunoprecipitated using Chip grade PU.1 or AICDA antibodies and respective pre-immune serum. 1% of pre-cleared chromatin was set aside as input control, de-cross linked and processed along with the immunoprecipitated fractions before subjecting to real time PCR. Quantitative PCR results were calculated using the SABioscience EpiTecChIP q PCR Data Analysis Template (http://www.sabiosciences.com/chippcrarray_data_analysis.php) and presented as % Input values.

### Cloning of mouse KLF4 Promoter

The mouse KLF4 promoter region −1481 to +45 bp relative to the transcription start site was PCR-amplified from genomic DNA using primers listed in Supplement S1. PCR product was analyzed by agarose gel electrophoresis and cloned into the pcr 2.1 TOPO vector at *SacI* and *EcoRV*. The PCR insert was released by digestion with *SacI* and *SmaI* and subcloned in to pGL3 yielding pGL3-KLF4(1.6 kb)-Luciferase. KLF4 insert was released from pGL3 using *SpeI*, *HindIII* and subcloned in to pcpgf-bas vector [31]. Nucleotide sequence of each construct was verified by DNA sequencing.

### Transient Transfection and Luciferase/mouse Alkaline Phosphotase (mSEAP) reporter Assays

An equal number (5–6×10^6^) of PU/ER(T) or HEK293 cells were electroporated with pGL3-KLF4 promoter constructs and pRLTK using Amaxa Electroporation kit. 36 h after transfection, cells were washed with cold PBS and lysed in 100 μl of Promega cell lysis buffer. Cell extracts were assayed for luciferase activity using a Promega dual luciferase assay system. Similarly, HEK293 cells were electroporated with pcpgf-KLF4 and grown in Invivogen HEK-Blue detection medium. mSEAP reporter activity was measured by absorbance of the growth medium at 630 nm.

#### Bisulfite conversion, methylation specific PCR and sequencing

Total genomic DNA from PU/ER(T) or wild type and GADD45α-/- bone marrow derived macrophages at different stages of differentiation was isolated and treated with MethylCode Bisulfite conversion reagent. The bisulfite converted DNA was purified and sequenced using M1CpG specific primers used for methylation specific PCR, listed in Supplement S1. DNA sequence results were subjected to multiple alignment using BiQ Analyzer [32], CpG dinucleotide distribution was plotted as bubble chart with filled circles representing methylated cytosine and open circles with demethylated cytosine bases.

#### Methylation Specific PCR

Methylation Specific Primers for KLF4 and GATA2 Promoters were designed using MethPrimer program [33]. Bisulfite converted genomic DNA was PCR amplified using methylation specific primers and SYBR green reaction mix. The methylation index was calculated as the ratio of methylated DNA cp values to the sum of cp values of methylated DNA and unmethylated DNA. The methylated and unmethylated DNA specific primer sequences are listed in Supplement S1.

### FACS analysis

Single-cell suspension of PU/ER(T) (1–2×10^4^/sample) was washed and incubated on ice for 30 minutes with appropriate fluorescently labeled antibodies. Cells were analyzed on an FACS Vantage flow cytometer (BD Biosciences) where gating was set based on respective unstained cell population and isotype matching control staining. The data were analyzed with FlowJo software.

### Statistical analysis

All the experiments were repeated three times for consistent results and representative data sets for EMSA, WB and flow cytometry are presented. Statistical analyses were done using GraphPad-Instat software with Mann-Whitney U test and significance in differences is calculated. p≤0.05 was considered significant. Error bars represent Standard Deviation.

## Results

### PU.1 specifically binds to KLF4 promoter

**Figure 1:**
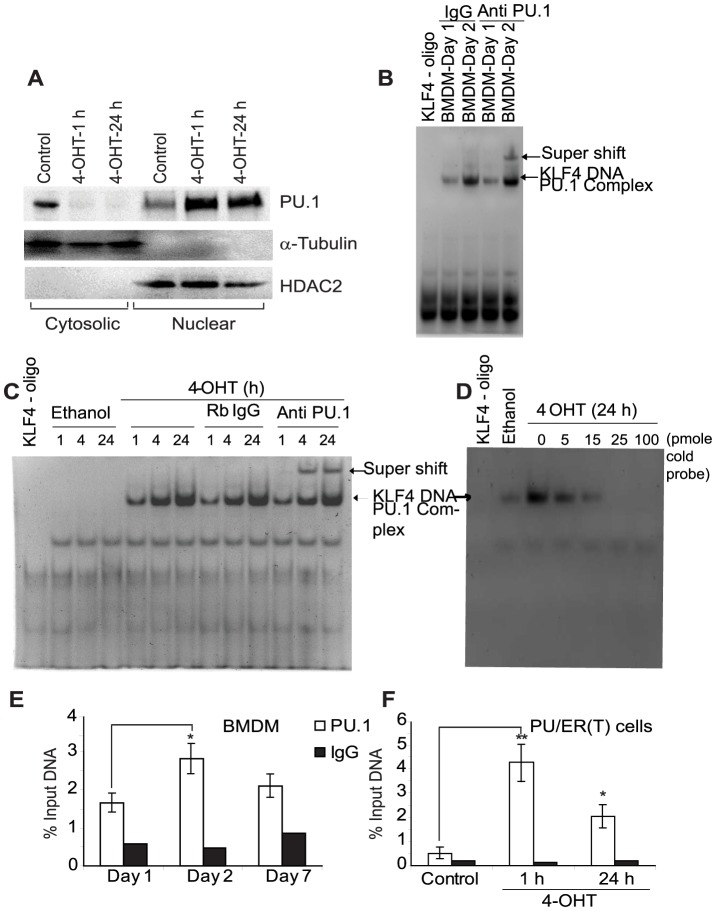
PU.1 binds specifically to KLF4 promoter during differentiation of myeloid progenitors to macrophages. **A**) PU/ER(T) cells were grown in presence of 95% Ethanol or 100 nM Tamoxifen for time periods as indicated. Cytosolic and nuclear protein fractions were prepared and translocation of PU.1 from cytosol to nucleus was detected by immunoblotting with anti PU.1 rabbit polyclonal antibodies. Purity of cytoplasmic and nuclear fractions was analyzed by immunoblotting with α-Tubulin and HDAC2 proteins. **B**) Nuclear extracts were prepared from differentiating bone marrow derived macrophages on day 1 and 7 and *in vitro* binding to biotin labeled 76 bp oligonucleotide probe representing −118/−113 PU.1 binding element in the KLF4 promoter was determined by EMSA as described in methods. **C**) PU/ER(T) cells were treated with Ethanol or 100 nM Tamoxifen for 1, 4 or 24 h and nuclear extracts were prepared. An equal amount of nuclear protein extract from Ethanol and Tamoxifen treatment was analyzed for DNA binding activity using biotin labeled 76 bp oligonucleotide probe representing −118/−113 PU.1 binding element in the KLF4 promoter. Specificity of PU.1-KLF4 DNA complex in experiment B & C was determined by the ability of the PU.1-KLF4 DNA complex to form supershift with anti PU.1 antibody. **D**) Specificity of PU.1-KLF4 DNA complex was determined by including 5, 15, 25 and 100 picomole of excess unlabelled KLF4 promoter oligo in the DNA binding reaction. **E**) *In vivo* binding of PU.1 to KLF4 promoter was determined by Chromatin-immuno precipitation in differentiating BMDM on day 1, 2 and 7 and in **F**) PU/ER(T) cells treated with Tamoxifen for 1 and 24 h. Error bars represent standard deviation and * is used where ever p value is ≤0.05 and ** is used when p≤0.005.

We used PU/ER(T) cell line generated from fetal liver cells of PU.1-/- mice that shows conditional PU.1 dependent macrophage differentiation [30], [34]. In these mice the wild type PU.1 gene is knocked in by 2 alleles of the PU/ER(T) transgene that was constructed by fusion of the modified estrogen receptor ligand binding domain-G525R, inducible by tamoxifen. In the presence of tamoxifen, the PU/ER(T) fusion protein is translocated into nucleus and becomes functionally active similar to WT PU.1, but in the absence of tamoxifen the fusion molecule remains in the cytoplasm [34]. As seen in Figure 1A, PU.1 protein translocates completely in to nucleus with in 1 h on stimulation with 100 nM of the estrogen receptor ligand 4-Hydroxy Tamoxifen. KLF4 is one of the target genes of PU.1 and to determine if the translocated nuclear PU.1 protein modulates KLF4 transcriptionally, we analyzed the promoter sequence of KLF4 for PU.1 binding elements by TF search [35]. KLF4 promoter shows two PU.1 consensus binding elements spanning from −118 to −113 and −40 to −35 bp upstream of transcription start site. Binding of PU.1 to its consensus sequence in KLF4 promoter was determined by Electrophoretic Mobility Shift Assay (EMSA) using a biotin labeled 76 bp KLF4 promoter oligo harboring the −118/−113 PU.1 binding element. Nuclear protein extracts from differentiating wild type mouse BMDM and tamoxifen treated PU/ER(T) cells showed increased binding of PU.1 to KLF4 promoter oligo indicating possible transcriptional regulation of KLF4 by PU.1 (Figure 1 B & C). The specificity of PU.1 binding to KLF4 promoter consensus sequence was shown by super shift of the PU.1-KLF4 DNA complex by rabbit polyclonal PU.1 antibody (Figure 1 B lane 5; Figure 1 C lane 12, 13). Control Rabbit IgG failed to cause super shift of PU.1-KLF4 DNA complex (Figure 1 B lane 2 & 3; Figure 1 C lane 8, 9 & 10). Also, the specificity of KLF4-PU.1 binding was confirmed by a cold competition assay using unlabelled KLF4 promoter oligo that decreased the intensity of labeled KLF4-PU.1 complex when used in increasing concentrations (Figure 1 D, lane 4–7). Supporting these results we also observed increased binding of PU.1 to KLF4 promoter at −118 bp region in differentiating BMDM and PU.1 inducible cell line by Chromatin Immuno precipitation (ChIP) assay (Figure 1 E & 1 F).

### PU.1 selectively directs monocyte/macrophage differentiation

**Figure 2:**
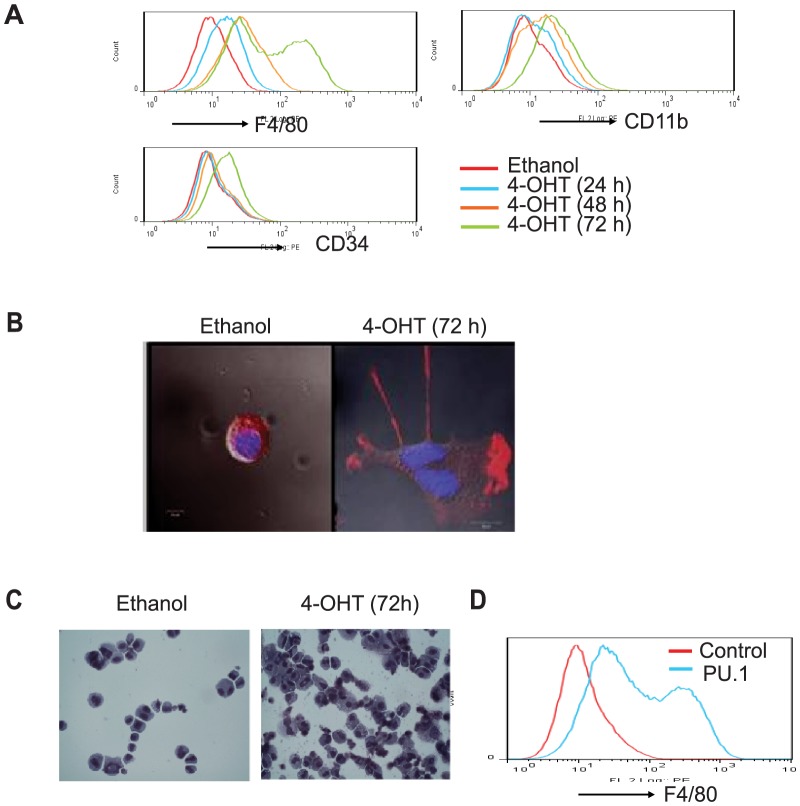
PU.1 directs the PU/ER(T) cells towards monocyte/macrophage differentiation. **A**) PU/ER(T) cells gown in presence of Tamoxifen or ethanol for 24, 48, 72 h and analyzed for surface expression of CD11b, F4/80 and CD34. **B**) PU/ER(T) differentiation to mature macrophages is determined by staining for PU.1 and Actin during pseudopod formation in tamoxifen treated cells. **C**) PU/ER(T) differentiation to mature macrophages is assessed by Giemsa staining after 72 h of Tamoxifen treatment. **D**) PU/ER(T) cells were electroporated with either PU.1 expression or control pCMV plasmids and analyzed after 72 h for surface expression of F4/80. Representative histograms of Flow cytometry or cell staining images from three different experiments are presented.

To test the effect of PU.1 on monocyte/macrophage lineage commitment and differentiation, PU/ER(T) cells were treated with 100 nM Tamoxifen up to 72 h and macrophage maturation is determined by cell surface expression of CD11b and F4/80, markers of mature macrophages (Figure 2 A). Population expressing CD11b and F4/80 significantly increased in a time dependent manner in tamoxifen treated PU/ER(T) cells. Pluripotent hematopoietic stem cell marker CD34 expression was found to slightly change at 72 h of differentiation of these cells (Figure 2 A). Tamoxifen treatment resulted in differentiation of myeloid progenitor cells towards mature macrophage phenotype as evidenced by pseudopod formation (Figure 2 B). Similarly, tamoxifen treatment caused morphological changes in PU/ER(T) cells like vacuolated cytoplasm and decreased nucleus to cytoplasmic size (Figure 2 C). More importantly, over expression of PU.1 cDNA plasmid alone in PU/ER(T) cells resulted in increased expression of F4/80 indicating the critical role of PU.1 in macrophage differentiation (Figure 2 D).

### KLF4 promoter undergoes demethylation during monocyte/macrophage differentiation

**Figure 3:**
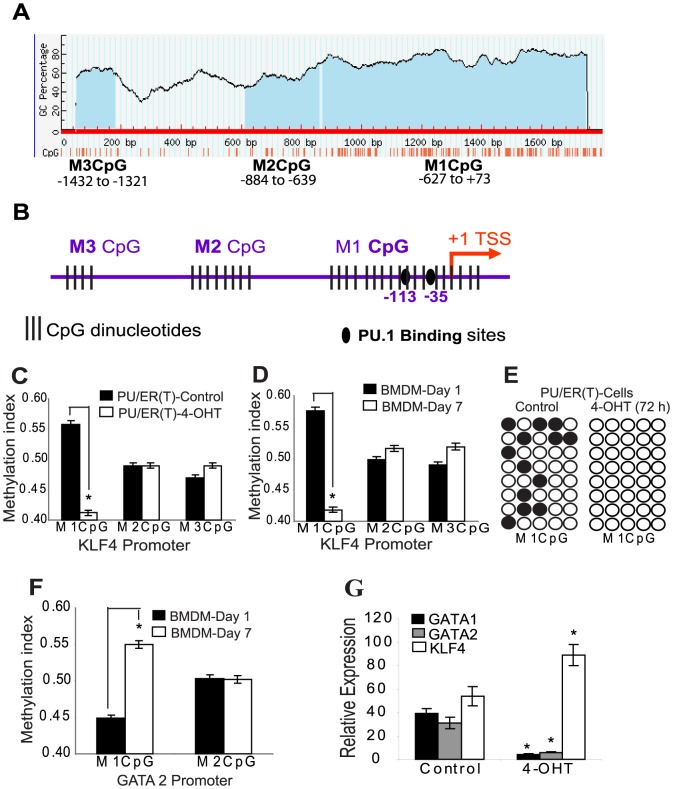
KLF4 promoter is demethylated during monocyte/macrophage differentiation. **A**) Methprimer analysis of KLF4 promoter indicating dense CpG methylation distributed as three distinct regions, M1CpG proximal to the transcription start site and two distal M2 and M3CpG regions away from the transcription start site. **B**) TF search analysis of 1.6 kb KLF4 promoter indicating putative PU.1 binding elements at −113 and −35 bp region from the transcription start site. **C**) Total genomic DNA was isolated, Bisulfite converted and used as template for qPCR using methylation specific primers. The relative methylation index of M1, M2 and M3 CpG regions calculated as described in methods was compared in PUER(T) cells after treatment with Ethanol or Tamoxifen for 72 h and in **D**) differentiating BMDM on 1^st^ and 7^th^ days **E**) Methylation of M1CpG region was compared by bisulfite sequencing in control and 72 h 4-OHT treated PU/ER(T) cells. **F**) Methylation index of GATA2 promoter CpG region 1 and 2 was determined using methylation specific primers designed using Methprimer. **G**) PU/ER(T) cells were grown in presence of 95% Ethanol or 100 nM Tamoxifen for 24 h and total cellular RNA was isolated and reverse transcribed. Using the resulting cDNA as template relative expression levels of KLF4, GATA1 and GATA2 normalized to β-Actin were determined by gene specific primers and SYBR green reaction mix. Error bars represent standard deviation. Figure C & G * indicates p≤0.005 Tamoxifen treatment compared to control; Figure D & F * indicates p≤0.005 seven day differentiated BMDM compared to one day BMDM.

In silico analysis of KLF4 promoter using Methprimer [33] showed dense hypermethylation with CpG dinucleotides distributed spanning the 1.6 kb region (Figure 3 A & B). As we observed that PU.1 binds to the KLF4 promotor during macrophage differentiation, methylation status of KLF4 promoter DNA is an important factor to determine its accessibility to PU.1 binding. Methylation specific quantitative PCR primers were designed separately for the three CpG regions, arbitrarily designated as M3CpG (spanning −1432 to −1321), M2CpG (spanning −884 to −689) and M1CpG (spanning −627 to +73) using the MethPrimer tool [33]. Using the methylation specific primers we analyzed the methylation status of KLF4 promoter in both Tamoxifen inducible PU/ER(T) cell line and wild type BMDM during early and late differentiation stages by bisulfite sequencing and quantitative PCR. Interestingly, the M1CpG region most proximal to the KLF4 transcription start site is demethylated indicated by decreased methylation index and bisulfite sequencing during differentiation of BMDM and PU/ER(T) cells (Figure 3 C, 3 D & 3 E). However, the methylation status of distal M2 and M3CpG region was unchanged during the differentiation. It was also observed that the methylation index of M1CpG in GATA2 promoter increased in 7 day differentiated BMDM (Figure 3 F). Tamoxifen treatment decreased expression of GATA1/GATA2 and increased the expression of KLF4 mRNA levels in PU/ER(T) cells (Figure 3 G).

### PU.1 transcriptionally regulates KLF4 promoter

**Figure 4:**
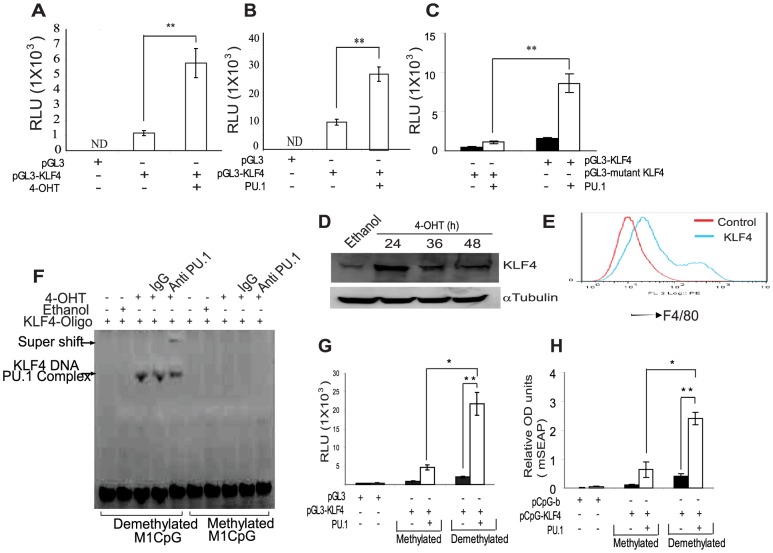
PU.1 transcriptionally regulates KLF4 expression that is sensitive to promoter methylation. **A**) PU/ER(T) cells were electroporated with 1.6 kb pGL3-KLF4 vector, after 16 h of electroporation treated with ethanol or 100 nM Tamoxifen for 24 h and analyzed for Luciferase reporter activity **B**) HEK293 cells were transfected with empty pGL3, 1.6 kb KLF4-pGL3, pCMV-PU.1 or pCMV control vectors separately or in combination as indicated in figure, and after 36 h cell extracts were prepared in Promega cell lysis buffer and analyzed for luciferase activity. **C**) HEK293 cells were transfected with empty pGL3, 1.6 kb mutant pGL3-KLF4, pCMV-PU.1 or pCMV control vectors separately or in combination as indicated in figure, and after 36 h cell extracts were prepared in Promega cell lysis buffer and analyzed for luciferase activity. **D**) PU/ER(T) cells were treated with Tamoxifen or ethanol for indicated time periods and expression of KLF4 was determined by immunoblotting. **E**) PU/ER(T) cells were electroporated with either control pCMV or KLF4 over expression vector and grown in IMDM for 72 h and analyzed for surface expression of F4/80. **F**) The biotin labeled KLF4 promoter oligo was methylated using *M.Sss*I in presence of S-Adenosyl Methionine and used as a probe to determine the DNA binding activity in the Ethanol and Tamoxifen treated nuclear extracts as described in figure 1. **G**) Empty pGL3 vector, pGL3-KLF4 Luciferase vector were in vitro methylated using *M.SssI* in presence of S-Adenosyl Methionine and electroporated along with control pCMV or pCMV-PU.1 expression vector. After 36 h, cell extracts were prepared in Promega cell lysis buffer and analyzed for luciferase activity. **H**) Empty pcpgf-basic, pcpgf-KLF4 were *in vitro* methylated using *M.sssI* in presence of S-Adenosyl Methionine and electroporated along with control pCMV or pCMV-PU.1 expression vector in to HEK293 cells and grown in HEK293Blue detection medium for 36 h. Relative promoter activity was compared by mSEAP reporter activity as measured by the absorbance of the HEK293 Blue growth medium at 630 nm. For figures 4 B, C, G, H reporter gene activities of pGL3-KLF4/pGL3-mutant KLF4/pcpg-KLF4 were not significantly different when co-transfected with the pCMV control plasmid. Figures 4 A to 4 C and 4 G, H means were compared and * is used where ever p value is ≤0.05 and ** is used when p≤0.005. Error bars represent standard deviation.

To analyze the effect of PU.1 on transcriptional regulation of KLF4 promoter, we cloned 1.6 kb of KLF4 promoter in to pGL3 Luciferase promoter probe vector and CpG free mSEAP reporter vectors [31]. PU/ER(T) cells transfected with pGL3-KLF4 vector showed increased Luciferase activity on Tamoxifen treatment (Figure 4 A). Similarly, co-transfection of PU.1 expression vector along with pGL3-KLF4 promoter vector in to HEK293 cells accentuated the promoter Luciferase activity (Figure 4 B) whereas the mutant pGL3-KLF4 showed attenuated reporter activity (Figure 4 C). Supporting these results, expression of KLF4 in tamoxifen treated PU/ER(T) cells increased during monocyte/macrophage differentiation (Figure 4 D) and over expression of KLF4 cDNA alone in PU/ER(T) cells increased surface expression of F4/80 similar to PU.1 (Figure 4 E).

### PU.1 Binding is specific to demethylated KLF4 promoter

To determine the effect of KLF4 promoter methylation on PU.1 binding, we *in vitro* methylated the CpG dinucleotides in the biotin labeled 76 bp KLF4 promoter oligo sequence by *M. SssI* in presence of S-Adenosyl Methionine and used as a probe in DNA binding assay. Tamoxifen treatment increased PU.1 binding to KLF4 promoter whereas this binding was completely abolished when the KLF4 promoter was *in vitro* methylated (Figure 4 F, lanes 3 to 5 versus 8 to 10). *In vitro* methylation of KLF4-pGL3 promoter probe vector by *M. SssI* completely abolished the KLF4 promoter reporter gene activity (Figure 4 G). The contribution of CpG residues in pGL3 vector back bone during *in vitro* methylation experiment was ruled out by using KLF4 promoter cloned in pcpgf-bas (Invivogen), CpG free mSEAP reporter vector with no CpG dinucleotides in the pGL3 vector back bone [31]. pcpgf-KLF4 construct also showed similar attenuation of promoter activity when *in vitro* methylated (Figure 4 H). This data consistently proved that demethylation of KLF4 promoter is essential for effective binding of PU.1 and subsequent transcriptional regulation of KLF4.

### Expression of Active demethylation proteins in PU/ER(T) cells

**Figure 5:**
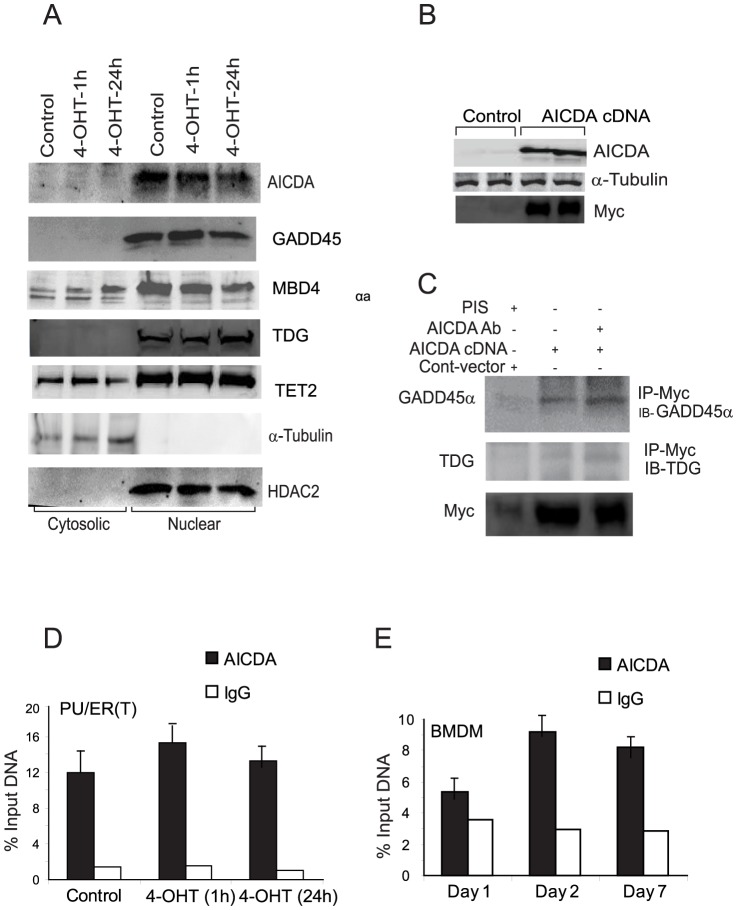
Identification of the active demethylase involved in KLF4 promoter demethylation. **A**) PU/ER(T) cells were grown in presence of Ethanol or 100 nm Tamoxifen for the indicated time periods and cellular localization of AICDA, GADD45α, MBD4, TDG and TET2 was determined by immunoblotting with respective antibodies in cytosolic and nuclear protein fractions as described in figure 1A. **B**) AICDA was over expressed by electroporation of AICDA or Control plasmids in to PU/ER(T) cells and after 24 h of electroporation total AICDA and Myc in cell lysates was detected by immunoblotting. **C**) Under similar conditions total AICDA in cell lysates was immunoprecipitated with anti myc Rabbit polyclonal antibodies and analyzed for co-immunoprecipitation of GADD45α, MBD4, TDG and TET2 by western blotting with respective antibodies. **D**) Recruitment of AICDA to KLF4 promoter was assessed by ChIP in Ethanol or Tamoxifen treated PU/ER(T) cells and **E**) differentiating bone derived marrow macrophages on Day 1, 2 and Day 7.

Next, we aimed to identify the mechanism of KLF4 promoter demethylation during monocyte/macrophage differentiation and if the proteins AICDA, GADD45α, MBD4, TDG or TET2 are involved in this process. Expression and localization of these proteins in cytosolic and nuclear compartments was determined by western blotting in differentiating PU/ER(T) cells treated with 100 nM tamoxifen. AICDA, GADD45α, MBD4, TDG or TET2 were expressed constitutively in PU/ER(T) cells and are localized predominantly in the nuclear fraction of tamoxifen treated PU/ER(T) cells (Figure 5 A). As AICDA is the proposed deaminase in GADD45α or TET mediated demethylation mechanisms, we investigated the role of AICDA in active demethylation of KLF4 promoter. Interestingly, AICDA was extensively studied in B-cells and its expression in myeloid cells was not reported. Therefore, AICDA mRNA expression was measured by quantitative RT-PCR in Tamoxifen treated PU/ER(T) cells and the RT-PCR product was further purified, sequenced and confirmed to be that of AICDA transcript (Supplement S1–H, NCBI nucleotide blast alignment).

### AICDA interacts with GADD45α and TDG

To test if AICDA interacts with the other putative demethylation proteins, we performed co-Immuno precipitation experiments. Myc tagged AICDA plasmid was over expressed by electroporation in PU/ER(T) cells and total cellular protein was immunoprecipitated with Anti-myc followed by immunoblotting for GADD45α, TDG, MBD4 and TET2 proteins separately. AICDA was found to interact with GADD45α strongly followed by TDG (Figure 5 C). MBD4 and TET2 were not detected in the AICDA immunoprecipitate (data not shown). Over expression of AICDA was confirmed by immunoblotting with AICDA and Myc antibodies (Figure 5 B).

AICDA might physically interact with KLF4 promoter DNA in the chromatin and this physical interaction may be direct or indirect involving protein factors implicated in demethylation process. The physical association of AICDA to the KLF4 promoter region was assessed by recruitment of AICDA on to the KLF4 promoter by Chromatin Immunoprecipitation (ChIP) assay using AICDA antibodies in differentiating BMDM and Tamoxifen treated PU/ER(T) cells (Figure 5 D & E). In both the cell types AICDA showed constitutive occupancy on KLF4 promoter M1CpG island region.

### AICDA is essential for KLF4 demethylation

**Figure 6:**
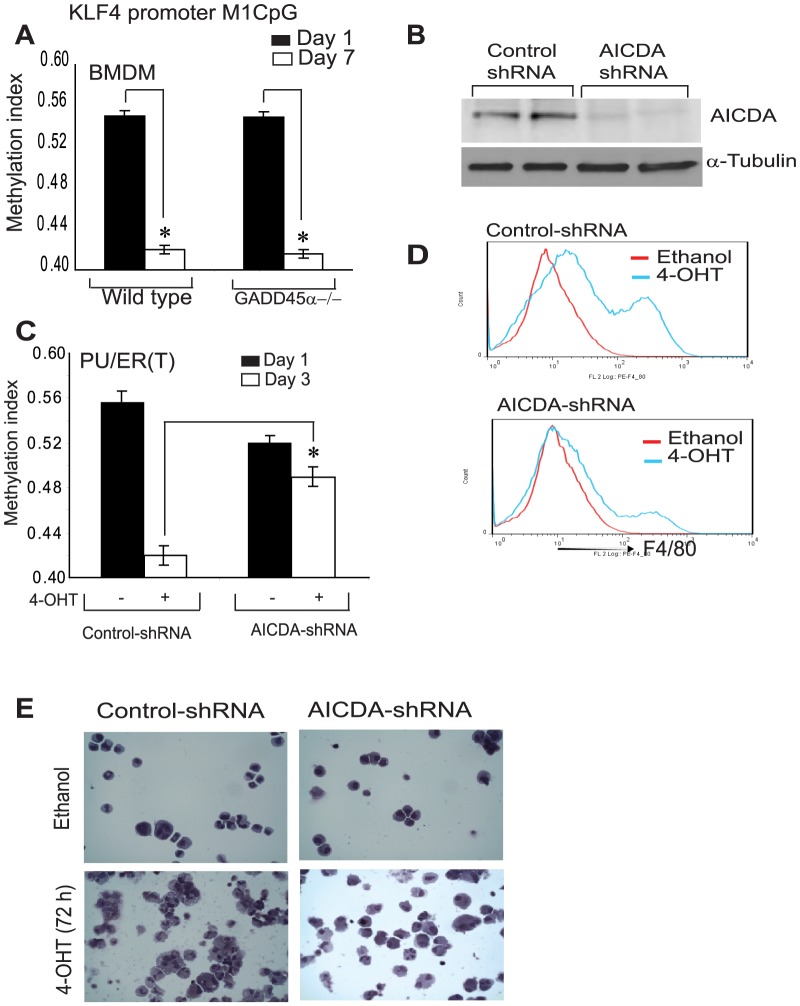
AICDA is essential for KLF4 promoter demethylation. **A**) The relative methylation index of the proximal M1 CpG region in KLF4 promoter was compared on 1^st^ and 7^th^ day of differentiating BMDM from wild type and GADD45α knock out macrophages as described in Figure 3 C&D. **B**) Knockdown of AICDA protein in AICDA-shRNA or control-shRNA electroporated PU/ER(T) cells. **C**) Methylation index of the proximal KLF4 promoter M1 CpG region was compared in AICDA shRNA or control shRNA electroporated PU/ER(T) cells as described in Figure 3C&D. **D**) Expression of F4/80 was analyzed in control shRNA or AICDA-shRNA electroporated PU/ER(T) cells treated with or without tamoxifen for 72 h. **E**) Morphological changes in ethanol/tamoxifen treated PU/ER(T) cells were analyzed in AICDA depleted cells in comparison with control cells. Representative histograms of flow cytometry, western blots and cell staining were presented. Figure 6A and C, * is used where ever p value is ≤0.05 and ** is used when p≤0.005. Error bars represent standard deviation.

Next, we determined the KLF4 promoter M1CpG region demethylation in differentiating GADD45α-/- and wild type bone marrow cells by methylation specific qPCR and bisulfite sequencing. Interestingly, in GADD45α-/- BMDM the KLF4 promoter was demethylated similar to wild type BMDM during differentiation, not supporting the requirement of GADD45α in KLF4 promoter sequence specific demethylation (Figure 6 A). Further, we investigated if AICDA deletion alters KLF4 promoter methylation in PU/ER(T) cells by using AICDA shRNA. PU/ER(T) cells were electroporated either with non specific control or AICDA shRNA plasmid (Origene). After 72 h of transfection the expression levels of AICDA were found to be reduced by more than 90% in AICDA shRNA transfected cells compared to control shRNA transfected cells (Figure 6 B). Methylation index of the M1CpG island in KLF4 promoter was quantitated by mehylation specific qPCR in PU/ER(T) cells electoporated either with control or AICDA shRNA. The AICDA shRNA transfected cells were differentiated in presence of tamoxifen or ethanol for 72 h before analysis. KLF4 promoter was demethylated in control shRNA transfected PU/ER(T) cells treated with tamoxifen whereas AICDA shRNA transfected cells failed to demethylate KLF4 promoter indicating a critical role for AICDA in active demethylation of KLF4 promoter (Figure 6 C). Next we determined if knock down of AICDA Expression has any effect on phenotypic characters of macrophage maturation. PU/ER(T) cells transfected with either control or AICDA shRNA were treated with ethanol or tamoxifen and expression of F4/80 was analyzed by flow cytometry and morphological characters by Giemsa staining. Control shRNA transfected cells treated with tamoxifen for 72 h showed increase in F4/80 positive cells compared to ethanol treatment (Figure 6 D) and mature macrophage cellular characters (Figure 6 E). AICDA knock down markedly attenuated the PU.1/KLF4 dependent increase in F4/80 positive population and mature macrophage morphological characters (Figure 6 D & E).

## Discussion

The critical role of PU.1 and KLF4 in monocyte/macrophage differentiation is well established [9]–[12]. In the present study, we addressed the question if PU.1 has any effect on chromatin status in terms of methylation changes during transcriptional activation of KLF4. The significant findings of the present study can be summarized as: 1) monocyte/macrophage lineage commitment of pluripotent fetal liver cells and hematopoietic cells is determined by PU.1 dependent KLF4 expression. 2) PU.1 binds to its consensus sequence in KLF4 promoter and this binding is associated with demethylation of KLF4 promoter. 3) Cloned KLF4 promoter Luciferase/mSEAP reporter vector showed increased Luciferase/alkaline phosphotase reporter activity in presence of PU.1 expression vector 4) PU.1 failed to bind *In vitro* methylated KLF4 promoter oligo and also showed attenuated KLF4 promoter reporter activity when it was *in vitro* methylated. 5) AICDA interacts with GADD45α and TDG but GADD45α was found to be dispensable for KLF4 promoter demethylation. 6) shRNA knock down of AICDA blocked demethylation of KLF4 promoter, F4/80 expression and macrophage differentiation. Together, these observations provide a mechanistic evidence for the role of AICDA in active demethylation/regulation of KLF4 expression during monocyte/macrophage lineage commitment and differentiation.

In the current study, using PU.1 inducible cells as monocyte/macrophage differentiation model, transcriptional regulation of KLF4 that is tightly controlled by its promoter methylation is established. In the same cellular system, we observed decreased expression of GATA1/GATA2 mRNA and maintenance of GATA2 promoter methylation during macrophage differentiation. The balance of PU.1 and GATA1/2 proteins supports the earlier literature, where GATA proteins are down regulated during myeloid lineage specification and their up regulation favors the cell fate towards erythroid lineage [36]. PU.1 is considered as the master regulator of different hematopoietic lineages and it regulates its target genes at multiple levels. During macrophage differentiation PU.1 associates with differentiation-associated epigenetic changes and binds to its target genes [37]. Binding of PU.1 was found to depend on its concentration, chromatin accessibility, PU.1 motif binding affinity and co-operative interaction with neighboring transcription factors [38]. This study also reported that PU.1 binding is independent of the genome wide DNA methylation where as our data identified a locus specific demethylation of KLF4 promoter which is required for PU.1 binding. Additionally, ChIP sequencing analysis during terminal differentiation of monocyte to osteoclasts, it was observed that PU.1 target genes undergo TET2 coupled demethylation and DNMT3b mediated methylation [39]. This study also showed that PU.1 interacts with both DNMT3b and TET2 and its knock down in primary monocytes impairs acquisition of DNA methylation marks and expression changes; reduced association of TET2 and DNMT3b at PU.1 target genes. We identified a similar role of PU.1 in associating with locus specific demethylation of KLF4 promoter and further attempted to investigate the proteins involved in demethylation.

Existing literature reported active DNA demethylation in several cell types including neurons, T-cells and human embryonic kidney cells [40], [41]. GADD45α promoted active DNA demethylation in zebra fish zygote, *X. laevis* oocytes and HEK293T cells [42]–[44]. However, several studies failed to substantiate the role of GADD45α in demethylation of DNA in mice [45], [46]. For example, neither global nor locus-specific methylation was increased in Gadd45α-/- mice [46], [29]. In the present study, we observed similar phenomenon where the KLF4 promoter in GADD45α-/- myeloid progenitors was not hyper methylated but underwent normal demethylation during monocyte/macrophage differentiation.

The role of another candidate protein of active demethylation, AICDA in DNA demethylation was initially postulated by Morgan et al [22] in inducing pluripotency in oocytes, embryonic germ cells and embryonic stem cells. Bhutani *et al*. [47] showed that expression of AICDA is required for promoter demethylation and induction of OCT4 and NANOG gene expression in interspecies heterokaryons of fused mouse embryonic stem cells and human fibroblasts. In another study, profiling of DNA methylation throughout the genome in wild-type and AICDA-deficient mouse Primordial Germ Cells (PGCs) revealed that AICDA-deficient PGCs were up to three times more methylated than wild-type ones [21]. Similarly, our study supported the role of AICDA in sequence specific demethylation of KLF4 promoter in differentiating myeloid progenitors. We found for the first time that AICDA was expressed in myeloid progenitor cells and found to physically associate with the KLF4 promoter. shRNA mediated suppression of AICDA resulted in blockade of maturation of myeloid progenitor cells in terms of morphological. cellular changes and F4/80 expression. Maturation of myeloid progenitors is mediated by transcriptional up regulation of KLF4 that resulted from active demethylation of its promoter by AICDA. To test if GADD45α was necessary for the sequence specific demethylation of KLF4 promoter, we compared the methylation of KLF4 promoter M1CpG region in differentiating macrophages from wild type and gadd45α knock out BMDM by methylation specific PCR. Interestingly, the gadd45α deficient macrophages did not show any defect in demethylation of the KLF4 promoter sequence, where as knock down of AICDA by shRNA drastically impaired the demethylation of KLF4 promoter and the morphological characters. In the present study, we observed a close correlation between demethylation of proximal CpG dinucleotides in KLF4 gene promoter, PU.1 binding, regulation of KLF4 expression and monocyte/macrophage differentiation. Our study postulated an important role of PU.1 binding to KLF4 promoter that depends on PU.1 binding affinity, and chromatin accessibility by changes in methylation status of KLF4 promoter DNA. Further investigations are required to determine the exact role of these site-specific methylation changes and other epigenetic modifications like histone methylation and or acetylation on macrophage differentiation. In summary, KLF4 promoter undergoes active DNA demethylation during monocyte/macrophage differentiation and AICDA plays an essential role in this demethylation process.

## Supporting Information

Supplement S1 **Supplement S1 contains primer sequences used for A) methylation specific qPCR B) Primers used for sequencing bisulfite converted genomic DNA C) Primers used for methylation specific PCR of GATA2 pomoter D) 5′ biotin labelled 76 bp KLF4 promoter oligo used for EMSA E) Primers used for cloning KLF4 promoter F) Primers used for KLF4 expression G) Primers used for AICDA expression H) Sequencing and confirmation of AICDA transcript from differentiating PU/ER(T) cells.**
(DOCX)
