# Supplementary material for: Krüppel Like Factor 4 Promoter Undergoes Active Demethylation during Monocyte/Macrophage Differentiation
Source: PLoS One. 2014 Apr 2;9(4):e93362. doi: 10.1371/journal.pone.0093362 (PMC3973678; doi:10.1371/journal.pone.0093362)
Supplement: Supplement S1 — contains primer sequences used for A) methylation specific qPCR B) Primers used for sequencing bisulfite converted genomic DNA C) Primers used for methylation specific PCR of GATA2 pomoter D) 5′ biotin labelled 76 bp KLF4 promoter oligo used for EMSA E) Primers used for cloning KLF4 promoter F) Primers used for KLF4 expression G) Primers used for AICDA expression H) Sequencing and confirmation of AICDA transcript from differentiating PU/ER(T) cells. (DOCX) [file pone.0093362.s001.docx]

**I. DNA Oligo sequences used in the study:**

**A. Primers used for methylation specific qPCR of KLF4 promoter:**

M1CpG island:

1. Klf4-met-fp1 5’ ATTTCGGGATTTAAATAGGTGAAAC 3’

2. Klf4-met-rp1 5’ CCTAACGAAAACAACTAAAAACGAA 3’

3. Klf4-unmet-fp1 5’ TTTGGGATTTAAATAGGTGAAATGA 3’

4. Klf4-unmet-rp1 5’ CCCTAACAAAAACAACTAAAAACAAA 3’

M2CpG island:

5. Klf4-met-fp2 5’ ATTTTTGGTGAAAGGTATTTTCGC 3’

6. Klf4-met-rp2 5’ AAAAACGACCGAAATTAAACACGTA 3’

7. Klf4-unmet-fp2 5’ GAGATTTTTGGTGAAAGGTATTTTTGT 3’

8. Klf4-unmet-rp2 5’ AAAAAAACAACCAAAATTAAACACATA 3’

M3CpG island:

9. Klf4-met-fp3 5’ ATTTTCGAGTTTAGGGAATCGATC 3’

10. Klf4-met-rp3 5’ TAACGAACTAAAACCGTACTAACGC 3’

11. Klf4-unmet-fp3 5’ TTTTTGAGTTTAGGGAATTGATTGT 3’

12. Klf4-unmet-rp3 5’ ACAACTAACAAACTAAAACCATACTAACAC 3’

**B. Primers used for sequencing bisulfite converted genomic DNA:**

1. Klf4-met-fp3 5’ ATTTTCGAGTTTAGGGAATCGATC 3’

2. Klf4-unmet-fp3 5’ TTTTTGAGTTTAGGGAATTGATTGT 3’

**C. Primers used for methylation specific PCR of GATA2 promoter:**

1. gata2-met-fp 5’ TTAGGTAGATAGGGCGTAGAGTTCG 3’

2. gata2-met-rp 5’ ACTATCTCTCAATTCCCGACCG 3’

3. gata2-unmet-fp 5’ GGATTAGGTAGATAGGGTGTAGAGTTTG 3’

4. gata2-unmet-rp 5’ TAACTATCTCTCAATTCCCAACCAC 3’

**D. KLF4 Promoter 76 bp oligo used for EMSA:**

5’-biotin-CGCGCAGTGGGGGCTGCGGGAAGGCGGGGAGAAGAAAGGCAGGGGGCGGGGCCTGG

CGGCGGAGCCCCGCGCGCCG-3’

**E. Primers for cloning KLF4 Promoter:**

Klf4-Pclonfp: 5’ GGTCCCAAGGATTCCGGGACTCAA 3’

Klf4-Pclonrp : 5’ CAGGTGAGAATGGCCGCGGTGG 3’

**F. Primers for KLF4 expression by RT-PCR:**

KLF4 FP1: 5’ GTCAGCGACGCTCTGCTCCC 3’

KLF4 RP1: 5’ CGCCAACGGTTAGTCGGGGC 3’

**G. Primers for AICDA expression by RT-PCR:**

mAIDF1: CCTCTGCTACGTGGTGAAGAGGAGA

mAIDR1: GGCAGCCAGACTTGTTGCGAAGGT

**H. Confirmation of PCR amplified AICDA mRNA transcript by sequencing:**
